# Sepax-2 cell processing device: a study assessing reproducibility of concentrating thawed hematopoietic progenitor cells

**DOI:** 10.1186/s12967-022-03703-1

**Published:** 2022-11-03

**Authors:** Bechara Mfarrej, Olivier Vicari, Sarah Ouffai, Carine Malenfant, Angela Granata, Sophie Thevenet, Christian Chabannon, Claude Lemarié, Boris Calmels

**Affiliations:** 1 Centre de Thérapie Cellulaire, Institut Paoli-Calmettes, Marseille, France; 2 Module Biothérapies du Centre d’Investigation Clinique de Marseille, AP-HM, Aix- Marseille Université, Institut Paoli-Calmettes, CBT-1409 Inserm, Marseille, France; 3 Aix-Marseille Université Faculté des Sciences Médicales et Paramédicales, Marseille, France

**Keywords:** DMSO, Hematopoietic progenitor cells, Sepax-2, Reproducibility, Cell therapy product

## Abstract

**Background:**

Autologous hematopoietic progenitor cell (HPC) transplantation is currently the standard of care for a fraction of patients with newly diagnosed myelomas and relapsed or refractory lymphomas. After high-dose chemotherapy, cryopreserved HPC are either infused directly after bedside thawing or washed and concentrated before infusion. We previously reported on the comparability of washing/concentrating HPC post-thaw vs. infusion without manipulation in terms of hematopoietic engraftment, yet settled for the prior favoring cell debris and DMSO removal. For almost two decades, automation of this critical step of washing/concentrating cells has been feasible. As part of continuous process verification, we aim to evaluate reproducibility of this procedure by assessing intra-batch and inter-batch variability upon concentration of thawed HPC products using the Sepax 2 S-100 cell separation system.

**Methods:**

Autologous HPC collected from the same patient were thawed and washed either in two batches processed within a 3-4 h interval and immediately infused on the same day (intra-batch, n = 45), or in two batches on different days (inter-batch, n = 49) for those patients requiring 2 or more high-dose chemotherapy cycles. Quality attributes assessed were CD34+ cell recovery, viability and CD45+ viability; CFU assay was only performed for allogeneic grafts.

**Results:**

Intra-batch and inter-batch median CD34+ cell recovery was comparable (75% vs. 73% and 77% vs. 77%, respectively). Similarly, intra-batch and inter-batch median CD45+ cell viability was comparable (79% vs. 80% and 79% vs. 78%, respectively). Bland-Altman analysis describing agreement between batches per patient revealed a bias close to 0%. Additionally, lower HPC recoveries noted in batch 1 were noted as well in batch 2, regardless of the CD34+ cell dose before cryopreservation, both intra- and inter-batch, suggesting that the quality of the collected product plays an important role in downstream recovery. Intrinsic (high mature and immature granulocyte content) and extrinsic (delay between apheresis and cryopreservation) variables of the collected product resulted in a significantly lower CD45+ viability and CD34+ cell recovery upon thawing/washing.

**Conclusions:**

Automated post-thaw HPC concentration provides reproducible cell recoveries and viabilities between different batches. Implications of this work go beyond HPC to concentrate cell suspension/products during manufacturing of cell and gene therapy products.

**Supplementary Information:**

The online version contains supplementary material available at 10.1186/s12967-022-03703-1.

## Background

Hematopoietic stem cell transplantation is currently the standard of care for a fraction of patients with first-line myelomas and relapsed or refractory lymphomas [1]. Cryopreservation of hematopoietic progenitor cells (HPC) is a routine step in the autologous setting and an optional or recommended step (for cord blood units for instance, or for other stem cell sources as recently experienced during the first waves of the COVID-19 pandemic) in the allogeneic setting [2, 3]. Nonetheless, cryopreservation poses challenges related to post-thaw cell viability and recovery as well as infusion-related adverse events [4].

We have previously reported on the comparability of thawing/washing/concentrating HPC vs. infusion after bedside thawing in terms of hematopoietic engraftment [5]. Automated processing of cryopreserved cells in the controlled environment of the processing facility does not compromise neutrophil reconstitution while providing additional benefits: concentrating HPC post-thaw allows for volume reduction, standardization, improved stability and ultimately a better risk-benefit profile of the graft infusion [6].

To that end, we used the Sepax 2 S-100 cell processing system (closed-system rotating syringe technology) for almost a decade in order to concentrate thawed autologous - and more recently allogeneic - HPC grafts, reduce dimethylsulfoxyde (DMSO) and cell debris in the formulated/infused cell product [7]. As part of our policy of continuous process verification, and consistently with JACIE requirements, we evaluated the reproducibility of this automated processing step by assessing intra-batch (2 cell processing runs of the same cell product performed on the same day) and inter-batch (2 runs of the same cell product on different days) variability. The goal is to gain certainty in the standardization process, upon repeated processing on the same material by different operators. Two situations allowed us to perform intra-batch evaluations: (a) poorly-mobilizing patients (following human granulocyte colony-stimulating factor) requiring more than one collection to reach the CD34^+^ target cell dose and (b) collections with relatively high TNC counts split into four bags for cryopreservation. Inter-batch variability was assessed in those patients who required two or more high dose chemotherapy cycles with autologous graft support.

## Methods

### Collection, cryopreservation and storage

Ninety four human granulocyte colony-stimulating factor–mobilized (r-hu-G-CSF) peripheral blood HPC products were collected and cryopreserved between July 2013 and January 2022. Patients consented to cell collection and transplantation as per institutional and EBMT policies. Patients and products characteristics are detailed in Table 1. After apheresis collection, platelet depletion and volume reduction were performed, followed by distribution into two EVA cryopreservation bags (Macopharma, France). Briefly, 50 mL of cryopreservation solution (6% hydroxyethyl starch, (Voluven, Fresenius Kabi, Germany) supplemented with 20% DMSO) was slowly added to 50 mL of cell suspension at + 4–10 °C to a final DMSO concentration of 10%, before introduction in a controlled-rate freezer (MiniDigitcool, IMV Cryo Bio System, France) and final storage in gas-phase liquid nitrogen tanks. All products tested negative for microbiological contamination (aerobic and anaerobic).

**Table 1:** Patients, donors and HPC products characteristics

Patients and donors characteristics	n (%)
allogeneic HSCT (RD)	12 (13%)
autologous HSCT	82 (87%)
Mobilisation	n (%)
rh-G-CSF	72 (77%)
rh-G-CSF + plerixafor	14 (15%)
missing data	8 (8%)
Nb of apheresis collections	n (%)
1	26 (28%)
2	68 (72%)
Nb of grafts processed	n
on same day	45
on different days	49
Median storage time (days)	n (min-max)
for bags infused on same day	42 (9-296)
for bags infused on different days	
1^st^ infusion	31 (10-141)
2^nd^ infusion	98 (41-190)

Abbreviations

HSCT : hematopoietic stem cell transplantation

RD : related donor

rh-G-CSF : recombinant human granulocyte colony-stimulating factor

### Thawing and cell processing

Thawing was performed using the Smart-Max (Cytiva Europe GmbH) medical device following our institutionally-validated thawing protocol: 9 min static + 1 min dynamic at + 37 °C. Post-thaw cell concentration was performed using the Sepax-2 S100 cell processing system (Cytiva Europe GmbH) using the SmartWash program: after a 1:1 dilution of the thawed bag with 6% hydroxyethyl starch, serial supernatant eliminations were conducted through automated centrifugation steps as were cell sedimentation and concentration of 2 bags into 1 final bag.

Two groups of processes, hereafter referred to as “runs”, were assessed: intra-batch thaw/wash sequence: same patient, same day cell-processing by the same operator at a 3–4 h interval (n = 45) and inter-batch thaw/wash sequence: same patient, different day cell-processing by a different operator (n = 49).

### Viable CD34^+^ cell enumeration and viability

Viable CD34^+^ cell count and viabilities (CD45^+^ and CD34^+^) were determined using an ISO 15189-accredited single platform flow cytometry assay. The method is based on the use of Stem-Kit reagents (Beckman Coulter, France) designed to comply with the modified International Society of Hemotherapy and Graft Engineering (ISHAGE, currently ISCT) protocol [8], which includes the use of Flowcount Fluorospheres (Beckman Coulter) for absolute viable cell counting. Sample acquisition was performed on Cytomics FC500 Flow Cytometer (Beckman Coulter). CD34^+^ cell recovery (%) was calculated as follows: ([post-wash viable absolute CD34^+^ cell count] / [pre-cryopreservation viable absolute CD34^+^ cell count]) x 100.

### Colony-forming unit assay

As a validated test for potency, an ISO 15189-accredited granulocyte-monocyte colony-forming unit assay (CFU-GM) assay was used for all washed allogeneic products, with semi-automated counting on STEM Vision (Stemcell technologies, USA) after a 14-day incubation period, as previously reported by our group [9]. Clonogenicity (%) is calculated as [number of CFU-GM colonies (×10^4^) / post-wash viable CD34^+^ cells (×10^6^)] x 100.

### Statistical analysis

Data were expressed as median and inter-quartile range (IQR). A two-tailed Mann-Whitney test was used when comparing two groups. Bland-Altman analyses were used to describe agreement between two runs. *p*-values ≤ 0.05 were considered significant. Statistical analyses were done using GraphPad Prism 5 (GraphPad).

## Results

### CD34^+^ cell recovery

To investigate the consistency of CD34^+^ cell recovery across different cell processing runs, we evaluated intra-batch runs and inter-batch runs. Intra-batch comparisons showed a median CD34^+^ cell recovery of 75% (IQR range: 66–83%) in the first run, vs. 73% (64–79%) in the second run, *p* = 0.54. Inter-batch runs had a median CD34^+^ cells recovery of 77% (IQR range: 68–85%) in the first run, and 77% (70–84%) in the second run, *p* = 0.74.

**Fig. 1:**
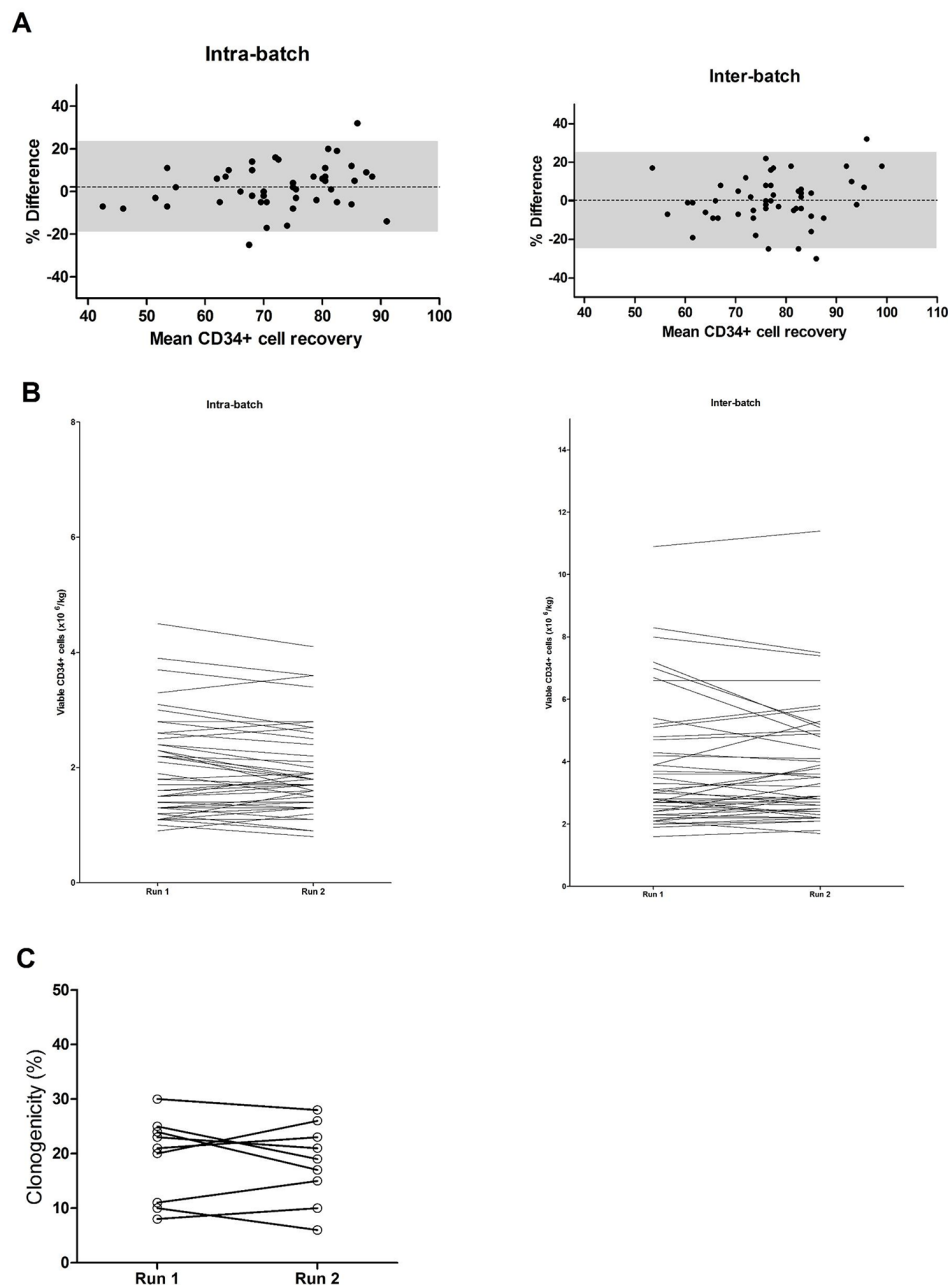
**Intra- (n = 45) and inter-batch (n = 49) differences in CD34**^**+**^**cell recovery and potency between runs.** Post-processing viable CD34^+^ cell dose was assessed by flow cytometry (modified ISHAGE now ISCT) and reported as CD34^+^ cell recovery compared to pre-cryopreservation dose. **(A)** Intra-batch and inter-batch CD34^+^ cell recovery data is plotted using a Bland-Altman plot. The y-axis represents the percent difference between 2 runs pertaining to the same patient vs. the mean CD34^+^ cell recovery between the 2 runs on the x-axis. The bias (agreement between both runs) is plotted as a dotted line, while the shaded area marks the 95% limits of agreement. **(B)** Intra-batch (left) and inter-batch (right) viable CD34^+^ cell doses are plotted by patient for each of the runs. **(C)** For 9 allogeneic grafts, a CFU-GM assay was performed via a 14-day culture followed by automated cell counting to report clonogenicity. Data are plotted per patient for each of the two runs pertaining to that patient

Our aim is to evaluate the agreement between the two situations (intra vs. inter-batches). Hence, we opted to statistically study the behaviors of the differences between one situation and the other. A Bland-Altman analysis was therefore performed to describe agreement between two measurements, quantifying the bias within which 95% of the differences between both measurements are included [10]. The differences were compared with the mean of the two paired values for two reasons: the need to evaluate the differences at different magnitudes of the measured variables and the fact that neither of the two measurements is a “reference value”. Intra- and inter-batch differences showed a bias +/- standard deviation of 2.2 +/- 10.7% for intra-batch and 0.3 +/- 12.6% for inter-batch. Inter-batch and intra-batch differences were homogeneous across different recoveries (50–100%) (Fig. 1 A).

CD34^+^ cells recoveries from the first and the second inter- and intra-batch runs were comparable, suggesting that recovery is mostly affected by the quality of the collected/cryopreserved product. (Fig. 1B). As per institutional policies, CFU-GM assays are only performed for allogeneic HPC grafts. Available data from the nine allogeneic products demonstrate comparable clonogenicity for both runs for the same patient (Fig. 1 C).

### CD34^+^ and CD45^+^ cell viabilities

We assessed viability of CD34^+^ and CD45^+^ cells in addition to CD34^+^ cell recovery. Intra-batch comparisons showed a median CD34^+^ cell viability of 78% (68–85%) and 78% (72–85%), respectively (*p* = 0.85), and a CD45^+^ cell viability of 79% (65–84%) and 80% (67–84%), respectively (*p* = 0.59). Inter-batch comparisons showed a median CD34^+^ cell viability of 84% (77–92%) and 85% (80–90%), respectively (*p* = 0.95) and a CD45^+^ cell viability of 79% (67–87%) and 78% (65–86%), respectively (*p* = 0.69).

**Fig. 2:**
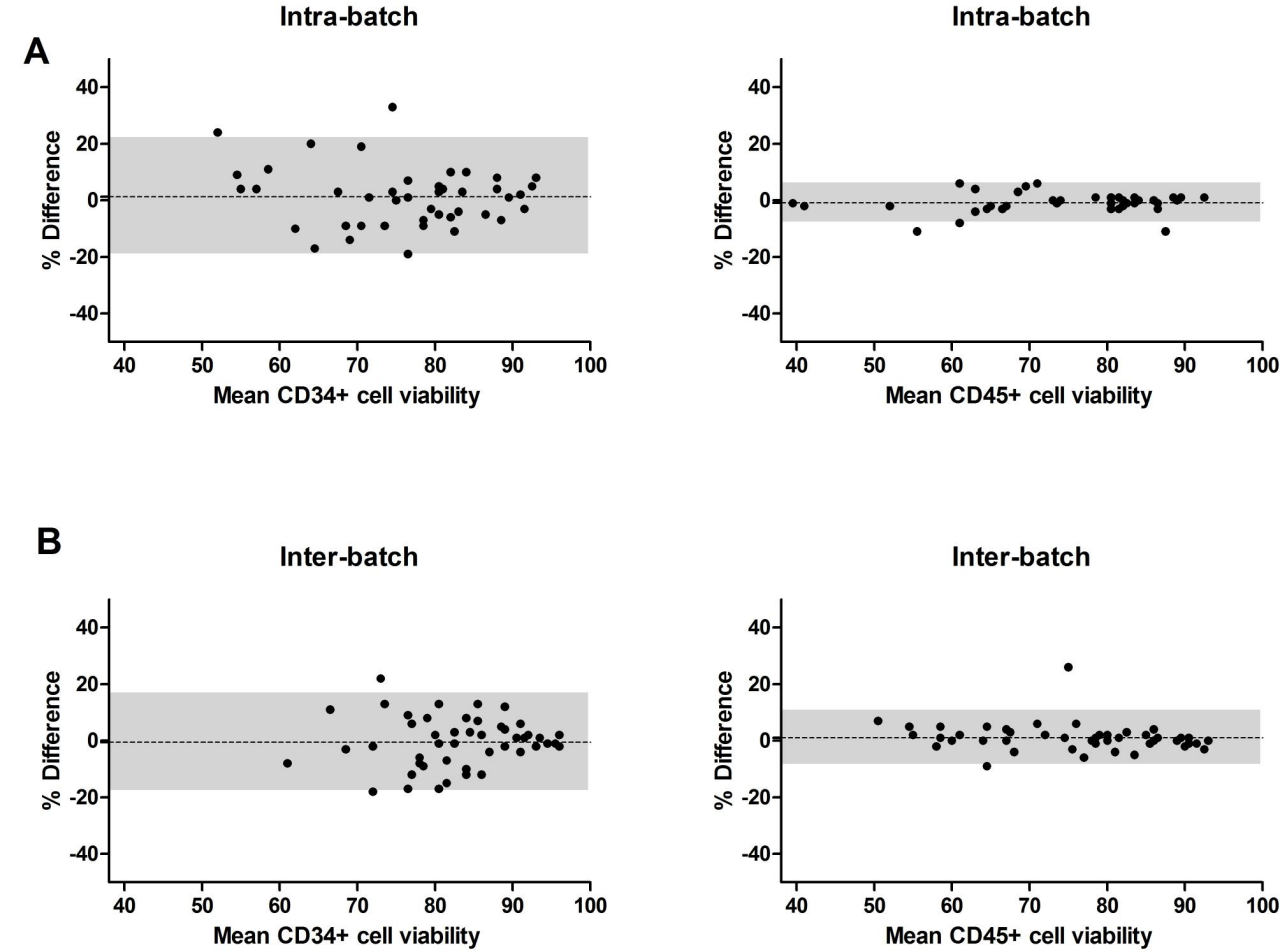
**Intra- (n = 45) and inter-batch (n = 49) differences in CD34**^**+**^**and CD45**^**+**^**cell viabilities.** Viabilities of CD34^+^ and CD45^+^ cells were performed by flow cytometry (modified ISHAGE) intra-batch (**A**) and inter-batch (**B**). CD34^+^ and CD45^+^ cell viability data are plotted using a Bland-Altman plot

The Bland-Altman analysis revealed intra- and inter-batch differences bias close to 0%: CD34^+^ cell viability intra-batch bias +/- standard deviation of 1.3 +/- 10.4% vs. inter-batch − 0.5+/- 8.8%; CD45^+^ cell viability intra-batch bias of -0.8 +/- 3.4 vs. inter-batch 1.1 +/- 4.8% (Fig. 2 A-B). A narrow 95% limit of agreement in CD45^+^ cell viability is noted in both comparisons across the range of viabilities 40–90%.

### Effect of product intrinsic and extrinsic variables on post-thaw recovery/viability

**Fig. 3:**
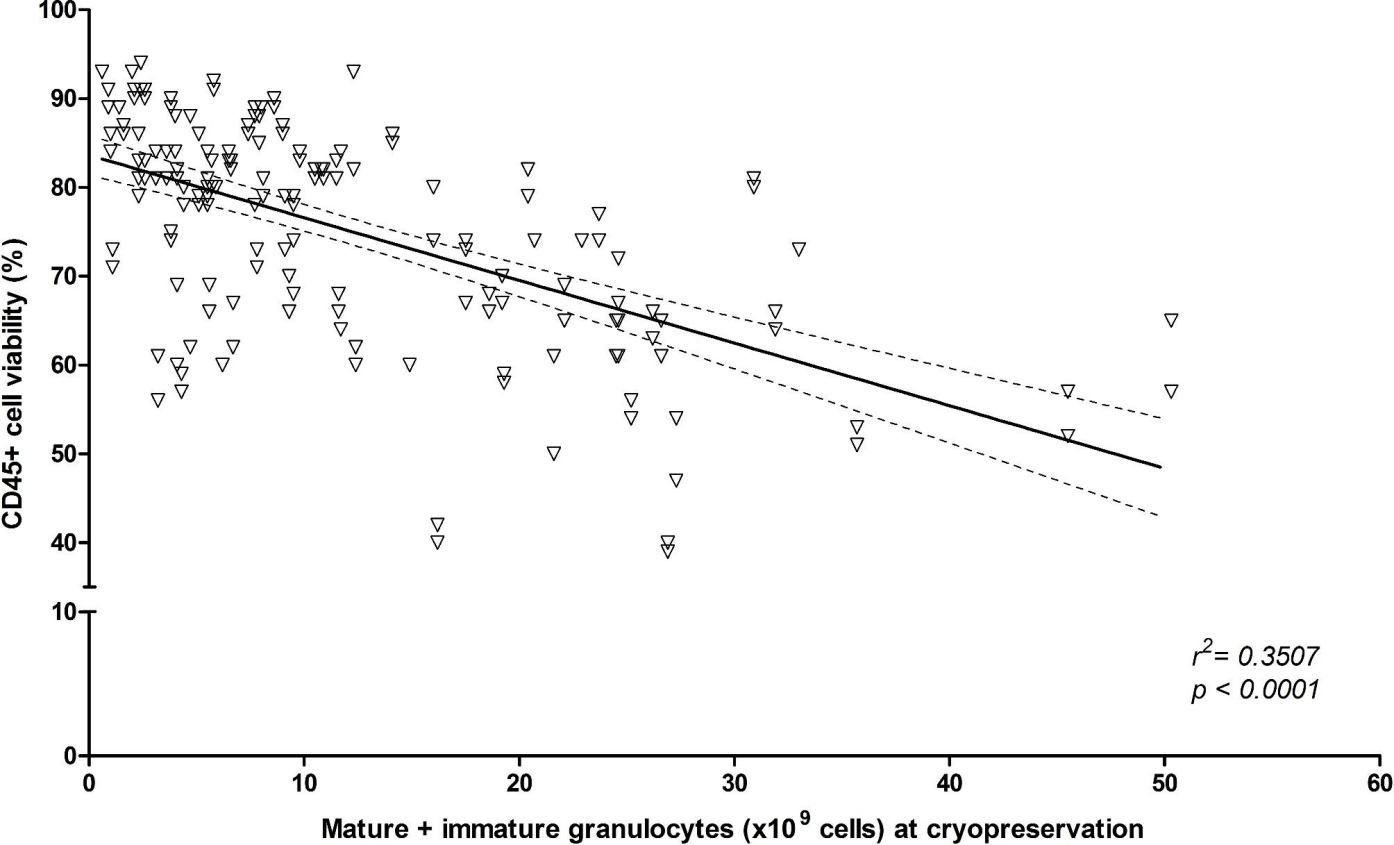
**Correlation between pre-cryopreservation granulocytes content and CD45 + cell viability post-processing.** Mature and immature granulocytes absolute counts are plotted against CD45 + cell viability. Best fit line (full line) for linear regression and 95% confidence intervals (dashed lines) are plotted. Goodness of fit and null hypothesis testing are shown as r2 and p value, respectively

Certain intrinsic and extrinsic variables related to the collected products could impact the quality of the final thawed/washed product, such as granulocytes contamination or delay between procurement and cryopreservation. We therefore first determined the impact of the mature and immature granulocytes content of the collected product on CD45^+^ cell viability post-thaw. A negative correlation was noted between the granulocytes absolute counts in the collected product and CD45^+^ cell viability post-processsing: *r*^*2*^ = 0.3507, regression coefficient − 0.7055 (± 0.0724), *p* < 0.0001 (Fig. 3).

**Fig. 4:**
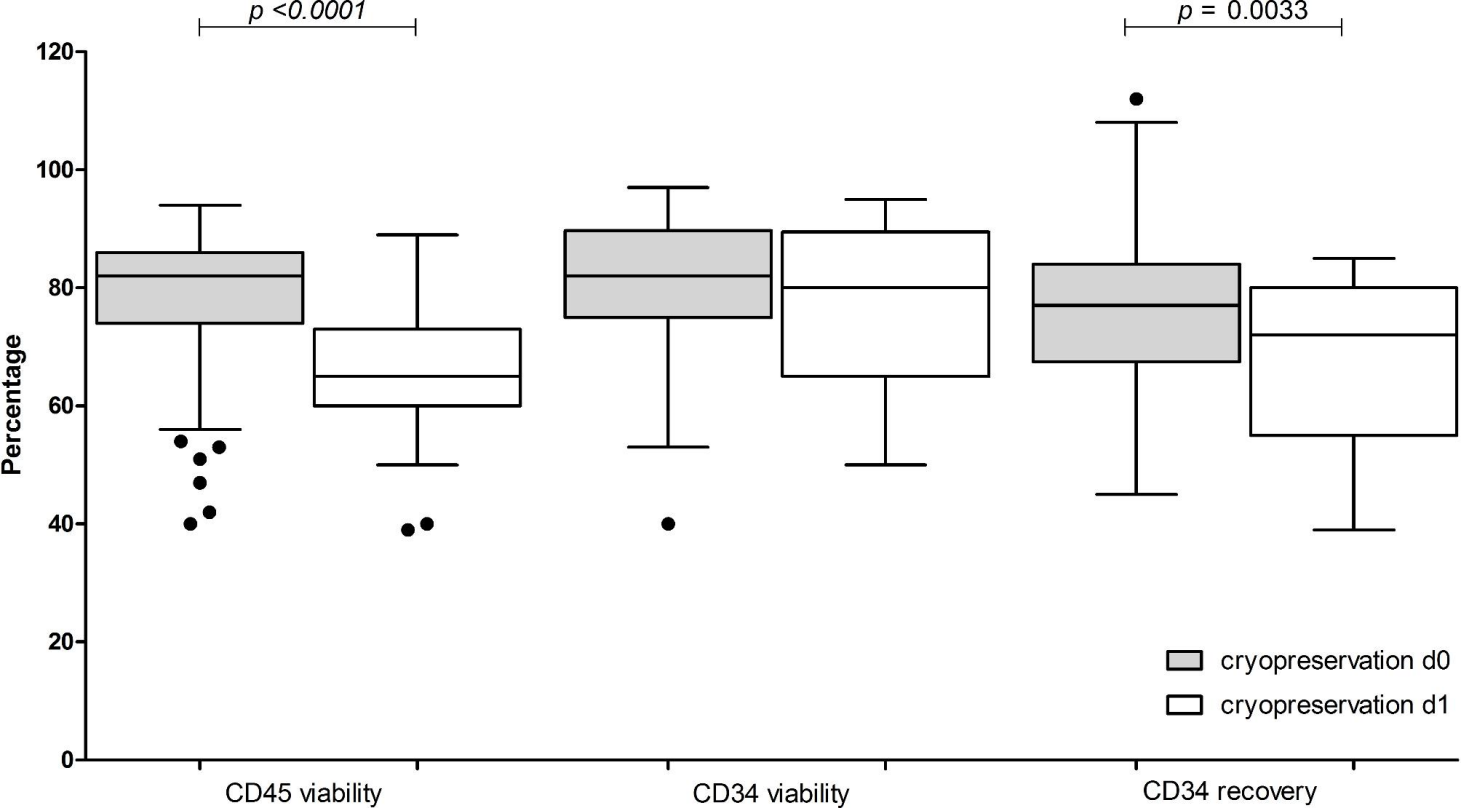
**Effect of delaying cryopreservation on post-thaw CD45**^**+**^**and CD34**^**+**^**cell viabilities and CD34**^**+**^**cell recovery.** Data pertaining to apheresis cryopreserved the same day of the collection (grey, d0) are compared to apheresis cryopreserved after overnight storage at + 4–10 °C (white, d1). Data is presented as box and whiskers, representing median with interquartile range, with whiskers representing min and max values, and outliers as dots. Variables compared are CD45^+^ viability, CD34^+^ cell viability and CD34^+^ cell recovery (compared to pre-cryopreservation). *p* values are reported above the groups using Mann–Whitney test for null hypothesis testing

Next, we investigated the effect of cryopreservation delay i.e. the time between the end of collection and start of controlled-rate freezing. Outcomes were CD45^+^ and CD34^+^ cell viabilities and CD34^+^ cell recovery of apheresis products cryopreserved the day of collection (d0) compared to those products stored overnight at + 4–10 °C before cryopreservation (d1) (Fig. 4). Significant differences (*p =* 0.0033) in CD34^+^ cell recovery (d0 median 77%, range 45–112% vs. d1 median 72%, range 39–85%, respectively) and CD45^+^ cell viability (d0 median 82%, 40–94% vs. d1 median 65%, 39–89%, *p <* 0.0001) were noted, despite TNC viability > 90% after overnight storage at + 4–10 °C.

## Discussion

Automation of the cell concentration steps post-thaw with the use of the Sepax-2 device was introduced in routine practice at our center in 2013, following an evaluation of the HPC minimal manipulation process and a risk-based analysis of the critical steps within it. This strategic move came about via a collaboration with the device manufacturer, aiming at improving process consistency, as is the consensus in the cell therapy field [11].

Initial performance qualification runs showed promising results and fitness of purpose for using Sepax-2 in concentrating thawed cells while removing > 95% of DMSO (*data not shown*). We hereby report on the reproducibility of the process of using Sepax-2 device over the past nine years, when used to serially wash and concentrate two thawed bags of 100 mL each into one final bag of 150mL. There was high agreement between runs using the same patient’s products, both intra- and inter-batch, in terms of CD34^+^ cell recovery, CD45^+^ and CD34^+^ cell viabilities. To the best of our knowledge, this is the first study to report on intra- and inter-batch CD34^+^ and CD45^+^ cell recovery and viabilities using automated cell processing. Overall, CD34^+^ cell recovery and CD45^+^ cell viability were comparable or slightly lower than those reported by other groups [4, 12]. Significantly inferior CD34^+^ cell recoveries were noted between our study and that of Sanchez-Salinas et al. [13]. This could be attributed to the use of the previous version of the Sepax, the S-100 (reported as being superior to Sepax-2 in terms of recovery [4]) and the CD34^+^ cell enumeration method that reports viability by Trypan Blue staining [14] and not viable CD34^+^ by single platform using ISHAGE guidelines. CD34^+^ and CD45^+^ cell viabilities, required as a quality control of the infused product by FACT-JACIE International Standards (8th edition), were comparable to other reports using autologous grafts [15].

We also show that conditions affecting the quality of the cell product, both intrinsic (granulocytes content) or extrinsic (delay between end of collection and cryopreservation) did not alter/modify the reproducibility of the process. We confirm that post-thaw CD45^+^ cell viability was negatively correlated with mature and immature granulocytes content of the collected product before cryopreservation, and that increasing the time between the end of collection and cryopreservation has also a detrimental effect on both CD45 viability and CD34 recovery. Delaying cryopreservation has been described as having a negative impact on cord blood-derived HPC viability [16]. We and others have previously reported on a negative correlation between the granulocyte content of the autologous infused graft and the occurrence and severity of adverse events [17, 18].

Our study has several strengths but also limitations. The first strength is the use of the process in unchanged conditions over nine years (program and kits did not change throughout this period). We consider that the use of a fully automated, operator-independent dry-thawing device allowing for reproducible thawing process of cryopreserved bags is a strength of our study, as compared to conventional water bath. Consistency of the analytical method used over time is another strength of this study [19, 20]. HPC functional assays on thawed allogeneic products showed reproducible clonogenicity between 2 runs, suggesting consistency of the post-thaw processing in terms of preserving CD34^+^ cell functionality. The comparability of CD34^+^ cell functionality was previously raised by Abonnenc et al. when assessing parallel or sequential washing of bags [21].

Based on the FDA’s guidance for industry, Process Validation: General Principles and Practices (cGMP, revision 1), we scrutinized intra-batch and inter-batch variations, as part of a continued process verification program. Maintenance of the facility, utilities and equipment, as well as auto-evaluation for operator skills, were not addressed in this study, despite being essential for a more comprehensive continued process verification program.

Reduced variability of intra-batch and inter-batch runs reported in our study gives confidence in the process and accommodates the variability in the incoming material. These findings have an impact in cell-based therapy processes, where fresh or cryopreserved starting materials require volume reduction and/or DMSO-removal post-thaw [22, 23] prior to proceeding to downstream manufacturing steps, like cell isolation [24, 25], washouts after activation and transduction or cell concentration and formulation [26, 27].

## Electronic supplementary material

Below is the link to the electronic supplementary material.

Supplementary Material 1

## Data Availability

The datasets analyzed are available from the corresponding author on reasonable request.
