# Supplementary material for: Sepax-2 cell processing device: a study assessing reproducibility of concentrating thawed hematopoietic progenitor cells
Source: J Transl Med. 2022 Nov 3;20:503. doi: 10.1186/s12967-022-03703-1 (PMC9632571; doi:10.1186/s12967-022-03703-1)
Supplement: Supplementary file 1 — Supplementary Material 1 [file 12967_2022_3703_MOESM1_ESM.pdf]

Reviewer #1

The article entitled "Sepax-2 cell processing device: a study of assessing reproducibility of concentrating thawed hematopoietic progenitor cells" is a very interesting article to those in the in the field of HSC transplantation. This article describes the use of a "new" automated and closed system instrument (Sepax-2) for washing and concentrating thawed HPC products, a process that is routinely performed at many cell manufacturing centers. This articles strengths are the relatively large number of grafts processed (n=94) and the detailed intra and inter-batch analysis of the products. Also, it was exciting to see the effect of delayed cryopreservation and the effect of grans on post thaw viabilities. Overall, this was an extremely well-written manuscript, the experiments backed up the conclusions stated, and the major findings were interesting and relevant to the broader community. I recommend acceptance of this article.

The authors thank reviewer #1 for these positive comments.

Reviewer #2

This paper shows overall benefits of the automation of cell therapy manufacturing. Dry thawing of cryopreserved product and automation of washing and concentrating of thawed autologous product can reduce operational cost, facilitate regulatory compliance and improve process consistency and lower contamination risk. Reducing variability of process and inter and intra batch data enhanced robustness, reliability and consistency of process.

Comment 1

In discussion (page 12, line 220) the authors mentioned that performance qualification showed Sepax-2 can concentrate thawed cells, while removing >95% of DMSO. How did the authors determine the removal % of DMSO? Was any specific technique used to measure DMSO concentration?

Comment 2

I would like to know why the authors are using Hydroxy Ethyl Starch based solution for washing HPC product? HES is contraindicated in severely ill patients presenting kidney failure or renal dysfunction. So why not 0.9% NaCl solution supplemented with albumin?

The authors thank reviewer #2 for these relevant remarks : clarifications are provided below.

Comment 1

DMSO quantification was performed using P/ACE capillary electrophoresis system (Sciex, Beckman Coulter), as described in [1] : 1 mL of thawed product was collected before and after washing on the Sepax-2 device. Percentage of DMSO elimination was calculated as (1 – [value after washing/value before washing])*100 = DMSO elimination %.

[1] Mfarrej B, Bouchet G, Couquiaud J, Regimbaud L, Binninger S, Mercier M, Lemarié C, Houzé P, Chabannon C, Calmels B. Pre-clinical assessment of the Lovo device for DMSO removal and cell concentration in thawed hematopoietic progenitor cell grafts. Cytotherapy. 2017 Dec;19(12):1501-1508. doi: 10.1016/j.jcyt.2017.09.001. Epub 2017 Oct 14. PMID: 29037941.

Comment 2

HES is routinely used at our institution since 2005. Since 2013, we are aware that use of HES is restricted. However, as stated in February 11th 2022 by the EMA's safety committee, PRAC, the marketing authorisations for HES solutions for infusion should be suspended across the European Union in order to minimise the risk of kidney injury and death in certain patients *i.e.* those critically ill, with burn injuries or with sepsis. Nor the type of patients or the volume of HES infused (plasma volume replacement following acute blood loss) can be compared to our routine use in HSC transplantation (no more that 250mL are infused in patients that are not critically ill, burned or with sepsis). However, given the 2022 statement of PRAC, we have now switched from HES to 0,9% NaCl solution supplemented with 5% HSA.
